# Stapling strategy enables improvement of antitumor activity and proteolytic stability of host-defense peptide hymenochirin-1B†

**DOI:** 10.1039/c8ra03446j

**Published:** 2018-06-19

**Authors:** Yulei Li, Minghao Wu, Qi Chang, Xia Zhao

**Affiliations:** Key Laboratory of Marine Drugs, Ministry of Education, School of Medicine and Pharmacy, Ocean University of China Qingdao 266003 China; Laboratory for Marine Drugs and Bioproducts, Qingdao National Laboratory for Marine Science and Technology Qingdao 266237 China 1184748799@qq.com

## Abstract

Hymenochirin-1B is a cationic, amphipathic, α-helical host-defense peptide with 29 residues, which was isolated from skin secretions of the Congo clawed frog and showed potent cytotoxic activities against a range of tumor cell lines. However, the application of hymenochirin-1B as a drug is limited due to its conformational flexibility and poor proteolytic stability. In this research, a series of hydrocarbon-stapled analogs of hymenochirin-1B were designed, synthesized, and tested. Some analogs showed remarkable improvement not only in α-helicity, but also in antitumor activity and protease resistance when compared to the parent peptide. The results indicated that most stapled peptide analogues possessed improved activities against a series of tumor cells; in particular, the bicyclic stapled peptide H-10 showed promising prospects for novel anti-tumor drug development. Our data demonstrated the important impacts of the all-hydrocarbon crosslink stapling strategy on the biological activity, proteolytic stability and helicity of hymenochirin-1B.

## Abstract 2

A series of stapled peptide analogs of hymenochirin-1B were efficiently prepared by an Fmoc-SPPS procedure. The peptide stapling strategy can improve the helicity, proteolytic stability and tumor cell-killing activity of linear peptide hymenochirin-1B.

## Graphical abstract

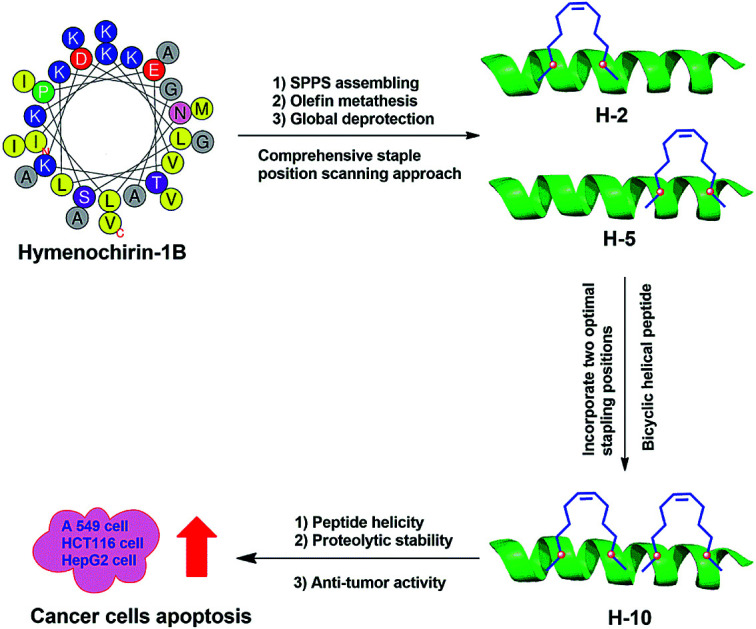

## Introduction

Cancer is still a major threat to human health and is the second leading cause of death worldwide. Almost one-sixth of deaths resulted from cancer, accounting for 8.8 million globally in 2015.^1^ At present, the treatments of cancer are chemotherapy, radiotherapy, surgery, immunotherapy and so on,^2,3^ but the overall survival rate of cancer patients still remains low. Therefore, it is urgent to discover more effective agents that either inhibit the growth or induce the apoptosis of tumor cells to improve the overall survival rate of cancer patients.

The hymenochirins are a family of α-helical host-defense peptides, which were first isolated from the skin secretions of the Congo clawed frog *Hymenochirus boettgeri* (pipidea).^4^ As a cationic, amphipathic, and α-helical host-defense peptide with 29 residues, hymenochirin-1B is the predominant pharmacological active component of four hymenochirins, which showed a wide of biological activities, such as antimicrobial,^5^ anticancer,^6^ immunomodulatory,^5^ and anti-diabetic activity.^7–9^ Previous studies of hymenochirin-1B and its analogues displayed potent cytotoxic activity against a range of tumor cell lines with appreciably less hemolytic activity against human erythrocytes.^6^ Despite its promising potential as a antitumor agent, hymenochirin-1B as a α-helical linear peptide has been limited by its inherent drawbacks. For example, native alpha-helical linear peptides usually do not retain their expected conformation and binding capability with the intended target,^10^ for they lack the structural reinforcement provided by the remainder of the protein. Moreover, the native linear peptides are also susceptible to proteolytic degradation, leading to the shortage of half-life period and bioavailability.

Peptide stapling is a strategy for constraining peptides typically in an α-helical conformation and improving proteolytic stability of linear peptides.^11^ Stapling is a peptide macrocycle which is performed by covalently linking the side-chains of two amino acids with Ru-catalyzed olefin metathesis,^12–15^ lactam-bridging,^16–18^ disulphides,^19^ triazoles,^20–22^ polyfluorobenzene^23,24^ and others. Among these strategies, Verdine and co-workers^12^ developed an all-hydrocarbon stapled strategy and has been considered as one of the most promising stapling strategy for protein–protein interactions (PPIs). The strategy has been applied in many native peptides to efficiently enhance the binding capability with intended target and protease resistance.^25–31^ Given all that, we speculated that the peptide stapling strategy would improve protease resistance and antitumor activity of host-defense peptide hymenochirin-1B. Therefore, a series of all-hydrocarbon stapled hymenochirin-1B analogs were generated with the Ru-catalyzed olefin metathesis, and their antitumor activities and stabilities were compared.

## Materials and methods

### 1. Materials

Fmoc-protected amino acids were purchased from Shanghai GL Biochem Ltd (shanghai, China). HCTU, HOBt, Oxyma were obtained from damas-beta (shanghai, China). Et_2_O, NMP, TFA, TFE, DMF, DCM, DIPEA, TIPS, piperidine, phenol, dimethylsulfoxide and other common reagent were purchased from Sinopharm Chemical Reagent Co. Ltd (shanghai, China). Purification of crude peptides was carried out by using HPLC LC-20AD SHIMADZU with dual wavelength (214 and 254 nm) (Japan). The Purity detection of peptides by using HPLC Thermo UltiMate 3000 Series (USA). Rink Amide MBHA resin (0.33 mmol g^−1^ loading) was purchased from Tianjin Nankai Hecheng Science & Technology Co. Ltd (Tianjing, China). Trypsin was purchased from TCI (Japan). All other commercially obtained reagents and solvents were used directly without further purification. Dulbecco's modified Eagle's medium (DMEM), fetal bovine serum (FBS), penicillin, and streptomycin were purchased from Gibco (Grand Island, NY, USA). The tumor cells A549, HepG2, HCT116 and normal cells LO-2, BEAS-2 293 T were obtained from the Cell Bank of Chinese Academy of Sciences (Shanghai, China), and cultured in DMEM supplemented with 10% FBS, 100 units per mL of penicillin and 100 μg mL^−1^ of streptomycin. All of the cells were incubated at 37 °C in an atmosphere of 5% CO_2_.

### 2. Peptide synthesis

#### 2.1 Synthesis of the linear template peptide hymenochirin-1B

##### Chain elongation

Rink Amide MBHA resin (350 mg, 0.33 mmol g^−1^ loading capacity) was swollen with dichloromethane (DCM, 5 mL) for 1 h in a fritted syringe. After removing the DCM, 20% v/v piperidine in dimethylformamide (DMF, 5 mL) was added to the beads in the syringe and allowed to shake on a tabletop shaker for 20 min twice at 37 °C. Following this, the resin was washed with DMF (3×, 5 mL), DCM (3×, 5 mL), DMF (3×, 5 mL), respectively. In a small glass vial, Fmoc–AA–OH (1 mmol), oxyma (1 mmol), diisopropylcarbodiimide (DIC, 1 mmol) and *N*-methyl pyrrolidone (NMP, 6 mL) were mixed and allowed to stand for 15 minutes at room temperature. The contents of the vial were then applied to the resin beads and allowed to shake for approximately 20 min at 60 °C. The resin was then sequentially washed thoroughly with DMF (3×, 5.00 mL), DCM (3×, 5.00 mL), DMF (3×, 5.00 mL), respectively, and then the resin was dried under a stream of air. The deprotection, coupling and washing steps were repeated until all the amino acid residues were sequentially installed to construct the linear peptide hymenochirin-1B.

##### Peptide cleavage from the resin and isolation

The peptide-bound resin was treated with 20% piperidine/DMF to remove the Fmoc group from the *N*-terminus, and the resin was dried under a stream of air. The beads were transferred to a small glass vial and brought up in the following cocktail: 5.0% v/v deionized water (0.75 mL), 2.5% v/v ethanedithiol (EDT, 0.375 mL), 5.0% v/v thioanisole (0.75 mL), 5.0% v/v phenol (0.75 mL) in 82.5% v/v trifluoroacetic acid (TFA, 12.375 mL). The resultant solution was allowed to shake for ∼3 h at room temperature. When completed, the cocktail was applied across the syringe frit, allowing the beads to be collected and discarded. The flow-through cocktail was collected in a polypropylene tube (∼15.00 mL), and TFA was evaporated by blowing with argon. The crude peptides were obtained by precipitation with 35 mL of cold diethyl ether and centrifugation at 3500 rpm for 3 min (3 times). The supernatant diethyl ether was decanted from the centrifuge tube and the crude peptides were allowed to air dry.

#### 2.2 Synthesis of the monocyclic stapled peptides

##### Chain elongation

The chain elongation of the monocyclic stapled peptide was carried out as described in the synthesis of the linear template peptide hymenochirin-1B. For couplings of S_5_/R_8_, Fmoc–(S_5_/R_8_)–OH (1 mmol), HATU (1 mmol), HOAT (1 mmol), DIPEA (1 mmol) and DMF (6 mL) were mixed for 1 min and then added to the resin at room temperature. After 1 hour, the resin was sequentially washed with DMF (3 times), DCM (3×, 5 mL), and DMF (3×, 5 mL). The deprotection, coupling and washing steps were repeated until all the amino acid residues were assembled.

##### Stapling of the peptide

The ring-closing metathesis reaction was carried out in 1,2-dichloroethane (DCE) at 35 °C using Grubbs' first-generation catalyst, the resin was washed with DCM (3×, 5 mL) and DCE (3×, 5 mL), and then treated with 10 mM solution of Grubbs' first-generation catalyst in DCE. After the first round of the 2 h metathesis, we repeated the same procedure for a second round of catalyst treatment with fresh catalyst solution, then the peptide-resin was washed with DMF (3×, 5 mL), DCM (3×, 5 mL).

##### Peptide cleavage from the resin and isolation

As described in the linear template peptide hymenochirin-1B, the monocyclic stapled crude peptides can be obtained after cleavage from the resin and isolation.

#### 2.3 Synthesis of the bicyclic stapled peptide

##### Chain elongation

The chain elongation of the bicyclic stapled peptide was carried out as described in the synthesis of the linear template peptide hymenochirin-1B. For couplings of S_5_, Fmoc–(S_5_)–OH (1 mmol), HATU (1 mmol), HOAT (1 mmol), DIPEA (1 mmol) and DMF (6 mL) were mixed for 1 min and then added to the resin at room temperature. After 1 hour, the resin was sequentially washed with DMF (3×, 5 mL), DCM (3×, 5 mL), and DMF (3×, 5 mL). The deprotection, washing, coupling and washing steps were repeated until the second Fmoc–(S_5_)–OH residue were assembled.

##### The first stapling of the peptide

The first stapling of the peptide was performed as described in the stapling of the peptide.

##### Chain elongation

The peptide-bound resin was treated with 20% piperidine/DMF to remove the Fmoc group from the *N*-terminus. For coupling of the next amino acid, Fmoc–AA–OH (1 mmol), oxyma (1 mmol), DIC (1 mmol) and NMP (6 mL) were mixed for 15 min, and then added to the resin at 60 °C. After 20 min, the resin was sequentially washed with DMF(3×, 5 mL), DCM (3×, 5 mL), and DMF (3×, 5 mL). For couplings of S_5_, Fmoc–(S_5_)–OH (1 mmol), HATU (1 mmol), HOAT (1 mmol), DIPEA (1 mmol) and DMF (6 mL) were mixed for 1 min, and then added to the resin at room temperature. After 1 hour, the resin was sequentially washed with DMF (3×, 5 mL), DCM (3×, 5 mL), and DMF (3×, 5 mL). The deprotection, washing, coupling and washing steps were repeated until the fourth Fmoc–(S_5_)–OH residue were assembled.

##### The second stapling of the peptide

The second stapling of the peptide was performed as described in the stapling of the peptide.

##### Chain elongation

The deprotection, coupling and washing steps were repeated until all the amino acid residues were sequentially installed to construct the bicyclic stapled peptide.

##### Peptide cleavage from the resin and isolation

The bicyclic stapled crude peptides can be obtained after cleavage from the resin and isolation as described in the linear template peptide.

### 2.4 Purification of peptides by reversed phase preparative HPLC

The target compounds were purified by the SHIMADZU (LC-6A) RP-HPLC. The purification was carried out using a C18 column (Daisogel, 20 × 250 mm) at a flow rate of 10 mL min^−1^. Buffer A consisted of acetonitrile with 0.1% TFA, while buffer B contained water with 0.1% TFA. The target compounds were purified by eluting with up to 75% buffer A in 50 minutes in a linear gradient, starting from 10% buffer A.

### 2.5 The Purity detection of peptide by reversed phase analytical HPLC

Analytical HPLC was run on Thermo Scientific Dionex UltiMate 3000 Series instrument using an analytical column (Boston pHlex ODS, 4.6 × 250 mM, 5 μm particle size, flow rate 1.0 mL min^−1^, r.t.). Analytical injections were monitored at 214 nm, 254 nm. Buffer A consisted of acetonitrile with 0.1% TFA, while buffer B contained water with 0.1% TFA. Analysis of the target compounds were carried out with up to 90% of buffer A in a linear gradient in 25 minutes, starting from 10% buffer A.

### 2.6 High resolution mass spectra

HR-Q-TOF-MS was measured on an Agilent 6530 Accurate Mass Q-TOF LC/MS mass spectrometer.

### 3. Circular dichroism

The linear and stapled peptides were dissolved in the trifluoroethanol (TFE) and water (1 : 1) to a final concentration of 50 μM, respectively. The CD spectra were obtained with 1 mm quartz cuvette on Jasco-815 spectropolarimeter at 25 °C. The measurement parameters were set up as follows: wavelength, 185–260 nm; step resolution, 0.5 nm; speed, 20 nm min^−1^. All spectra were converted to a uniform scale of molar ellipticity after background subtraction. The curves were smoothed using standard parameters. The ellipticity in the acquired CD spectra was converted to the molar ellipticity using the equation: [*θ*] = *θ*/(10 × *c* × *l*), where, [*θ*] is the Molar ellipticity (deg cm^2^. d mol^−1^), *θ* is the observed ellipticity corrected for the buffer at a given wavelength (m deg), *c* is the molar concentration and *l* is the path length (cm). The helicity of each peptide was calculated as described in a literature based on the ellipticity of the peptide's spectrum at 222 nm and the number of amino acids in the peptide sequence.

### 4. Growth inhibition of the peptides against tumor cells

Anti-tumor activity was evaluated by the standard MTT assay with a slight modification measuring the median inhibitory concentration (IC_50_) values.^35^ HCT-116 cells, HepG2 cells and A549 cells were plated in triplicate wells into a 96-well plate at 5 × 10^3^/well and cultured routinely for 24 h. Cells were incubated with increasing concentrations of hymenochirin-1B and its analogs (0.1–30 μM) and continued to culture with DMEM plus 10% FBS at 37 °C and 5% CO_2_. After being incubated for 96 h, the absorbance A value was determined by MTT at 540 nm. The untreated HCT-116 cells, HepG2 cells and A549 cells were used as control. Each experiment was performed in duplicate. The inhibitory rate was calculated according to the following formula: inhibitory rate = (a value of the control group − a value of the study group)/a value of the control group × 100%.

### 5. Protease resistance

The peptides were dissolved in PBS buffer solutions (50 mM, pH = 7.4) to a final concentration of 1 mM, respectively. Trypsin was dissolved in PBS buffer (50 mM, containing 2 mM of CaCl_2_, pH = 8) to a final concentration of 5 ng μL^−1^. Then the peptide solutions (100 μL) were incubated with trypsin solution (1 mL) at room temperature. 100 μL of digestion mixture was taken at the 0, 0.5, 1.0, 1.5, 2.0, 2.5, 3.0, and 3.5 h marks, then quenched with 20 μL of hydrochloric acid (1 M). The solution of the trypsin peptide fragments was monitored by HPLC at different time to determine the fraction of protease degradation at 214 nm. Each experiment was performed in duplicate.

## Results and discussion

### 1. Design and characterization of stapled peptides

**Fig. 1:**
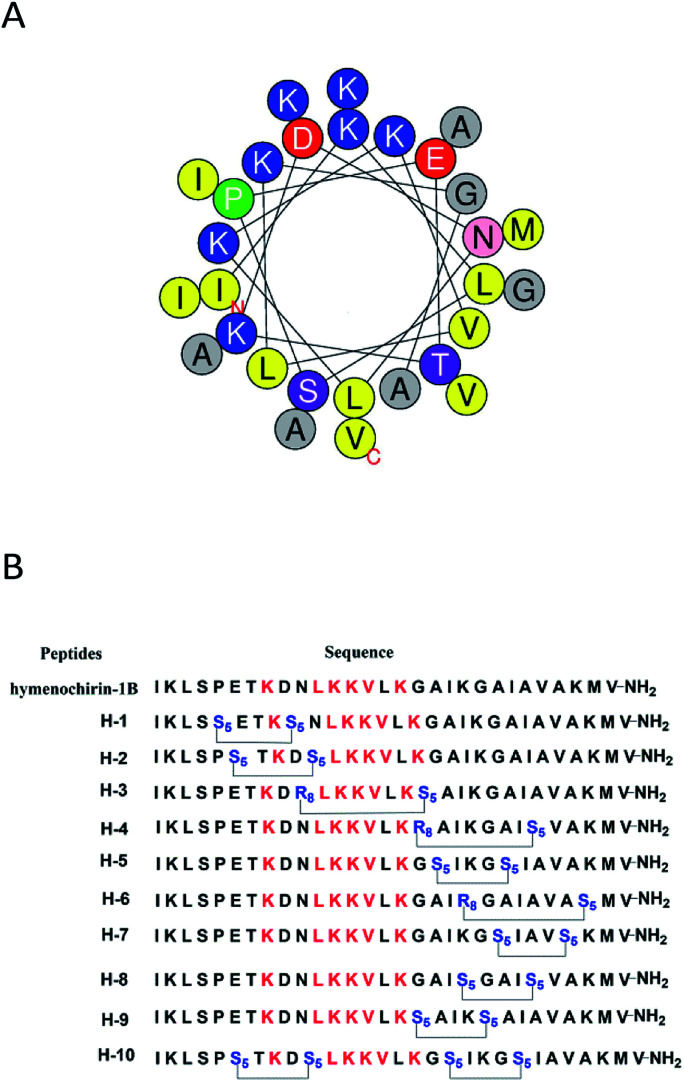
(A) Helical wheel presentation of the hymenochirin-1B. The yellow points to the hydrophobic residues. The blue points to the positively charged hydrophilic residues. The red points to the negatively charged hydrophilic residues. The purple points to noncharged polar residues. The gray represents other residues. The green points to the proline. (B) Sequence of stapled hymenochirin-1B analogs. The red represents the conserved residues. The blue points to the changed residues covalently linked by Ru-catalyzed olefin metathesis. S_5_ = (*S*)-*N*-Fmoc-2-(4′-pentenyl)alanine; R_8_ = (*R*)-*N*-Fmoc-2-(7′-octen-yl)alanine.

**Scheme 1:**
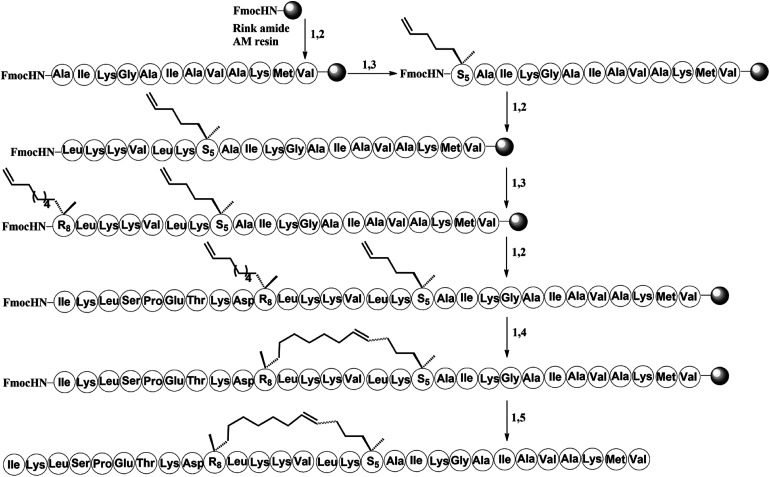
Solid-phase synthesis of stapled peptide H-3. Conditions: (1) 20% piperidine in DMF 5 min (2 times), 35 °C; (2) Fmoc–AA–OH (4 equiv.)/Oxyma (4 equiv.)/DIC (4 equiv.), 20 min, 60 °C; (3) Fmoc–S_5_/R_8_–OH (4 equiv.)/HATU (4 equiv.)/HOAT (4 equiv.)/DIPEA (4 equiv.), 60 min, 35 °C; (4) 6 mM 1st Grubbs', catalyst, DCE, 2 h, 35 °C; (5) reagent K (TFA : H_2_O : EDT : thioanisole : phenol = 82.5 : 5 : 2.5 : 5 : 5), 3 hours, 35 °C.

**Scheme 2:**
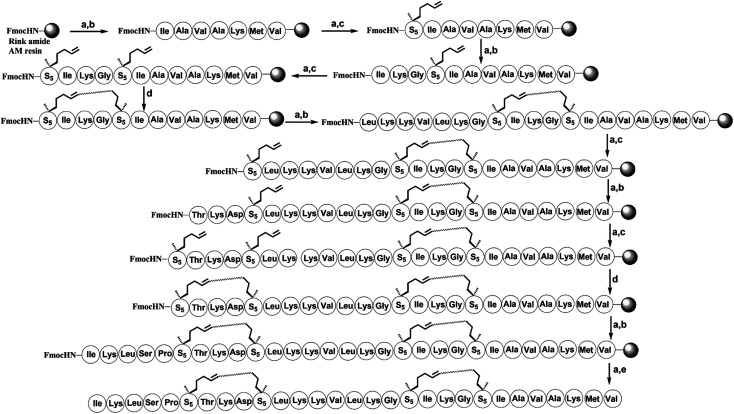
Solid-phase synthesis of bicyclic stapled peptide H-10. Conditions: (a) 20% piperidine in DMF 5 min (2 times), 35 °C; (b) Fmoc–AA–OH (4 equiv.)/Oxyma (4 equiv.)/DIC (4 equiv.), 20 min, 60 °C; (c) Fmoc–S_5_–OH (4 equiv.)/HATU (4 equiv.)/HOAT (4 equiv.)/DIPEA (4 equiv.), 60 min, 35 °C; (d) 6 mM 1st Grubbs', catalyst, DCE, 2 h, 35 °C; (e) reagent K (TFA : H_2_O : EDT : thioanisole : phenol = 82.5 : 5 : 2.5 : 5 : 5), 3 hours, 35 °C.

To investigate the influence of the all-hydrocarbon stapled strategy on the biological activity and protease resistance ability of hymenochirin-1B, ten all-hydrocarbon stapled hymenochirin-1B analogs were synthesized (Fig. 1B). Helical wheel projection indicated α-helix structure of hymenochirin-1B (Fig. 1A), of which Lys8, Leu11, Lys12, Lys13, Val14 and Lys16 were the conserved amino acid residues of hymenochirin-1B.^5^ We adopted a more comprehensive staple scanning approach, which essentially all staple positions were along the α-helical domain of hymenochirin-1B from residue 5 to 27 without replacing the conserved amino acid residues. The building blocks for all-hydrocarbon stapling are α,α-disubstituted non-natural amino acids bearing terminal olefin tethers of varying length. We investigated the macrocycle of different size to cross-link two non-natural amino acids and induce 1–2 helical turns in the peptide. For single turn stapling, we used (*S*)-*N*-Fmoc-2-(4′-pentenyl)alanine at *i*, *i* + 4 positions (Pro5/Asn9, Glu6/Asn10, Ala18/Ala22, Ala22/Ala26, Lys20/Ala24, Gly17/Gly21) to obtain peptides H-1, H-2, H-5, H-7, H-8, H-9, respectively. For double turn stapling, we employed a combination of (*S*)-*N*-Fmoc-2-(4′-pentenyl)alanine with (*R*)-*N*-Fmoc-2-(7′-octenyl)alanine at *i*, *i* + 7 positions (Asn10/Gly17, Gly17/Ala24, Lys20/Lys27) to achieve peptides H-3, H-4, H-6, respectively (Scheme 1). Through the screening of antitumor activity, the two optimal stapling positions (Glu6/Asn10, Ala18/Ala22) were determined. We schemed to incorporate the two optimal stapling positions into the templated peptide hymenochirin-1B to investigate whether the bicyclic helical peptide can further improve the anti-tumor activity. For bicyclic helical peptide, we used (*S*)-*N*-Fmoc-2-(4′-pentenyl)alanine at (Glu6/Asn10, Ala18/Ala22) to obtain H-10 (Scheme 2). Matrix-assisted laser desorption/ionization time-of-fight mass spectroscopy (MALDI-TOF-MS) was employed to confirm the molecular weight of peptide, and the measured molecular weight of each peptide was consistent with the theoretically calculated value (as listed in ESI†).

### 2. Synthesis of stapled peptides

**Fig. 2:**
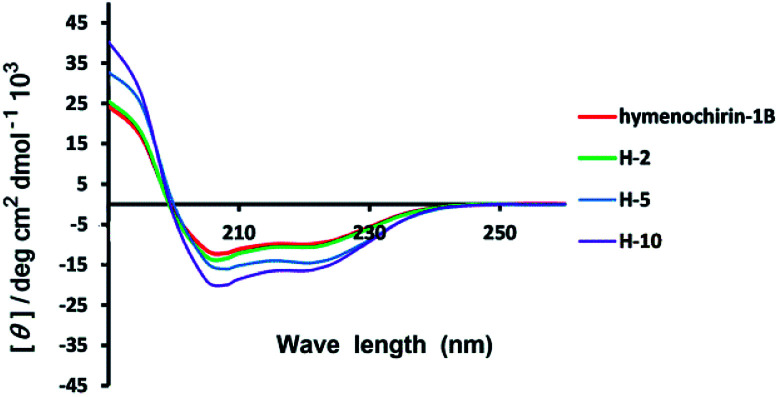
Circular dichroism spectra of linear model peptide hymenochirin-1B (in red), stapled peptide H-2 (in green), H-5 (in blue) and H-10 (purple). The peptides were dissolved in the PBS buffer at a final concentration of 50 μM. The percent of helicity was calculated based on the [*θ*]_222_ value.^34^

The all-hydrocarbon stapled peptide analogues H-1 to H-10 and the template peptide hymenochirin-1B were prepared using a Fmoc solid-phase peptide synthesis (SPPS) procedure with Rink Amide MBHA resin as the solid support (Schemes 1 and 2). Oxyma (ethyl 2-cyano-2-(hydroxyimino)acetate) was used as a coupling reagent, which was lower rate of racemization and more effective than other coupling reagents such as *O*-(7-azabenzotriazol-1-yl)-*N*,*N*,*N*′,*N*′-tetramethyluronium-hexafluorophosphate (HCTU) and 1-hydroxybenzotriazole (HOBt).^33^ (*O*-(7-azabenzotriazol-1-yl)-*N*,*N*,*N*′,*N*′-tetrame-thyluronium hexafluoro-phosphate) (HATU), 1-hydroxy-7-azabenzotriazole (HOAT), and *N*,*N*-diisopropylethylamine (DIPEA) were used for coupling of (*S*)-*N*-Fmoc-2-(4′-pentenyl)alanine and (*R*)-*N*-Fmoc-2-(7′-octenyl)alanine. After the linear peptides assembly were completed, the olefin-containing peptide was stapled using Grubbs' first-generation catalyst. The peptide was cleaved off from the resin and globally deprotected with reagent K (TFA : H_2_O : EDT : thioanisole : phenol = 82.5 : 5 : 2.5 : 5 : 5). Precooled diethyl ether was added to precipitate crude peptides, and then peptides were purified by semi-preparative RP-HPLC (Schemes 1 and 2) (Fig. 2).

### 3. Secondary structure of peptides

Circular dichroism (CD) spectroscopy was used to investigate the secondary structures of the stapled and template peptides to probe structural changes induced by conformation constraint in a membrane-like milieu, including trifluoroethyl (TFE) and water (1 : 1). CD analysis of the peptides demonstrated that the helicity of the template peptide hymenochirin-1B was only 48.7%, while the helicity of H-1 to H-10 range from 49% to 73.3% corresponding to a 1.1 to 1.5-fold increase. (Table 1, ESI†). These results demonstrated that the all-hydrocarbon stapled strategy can improve the helicity compared with the template peptide which is consistent with the previous reports.^32^

**Table 1:** The α-helicity and anti-tumor activity of stapled peptides

Peptide	Helicity (%)	IC_50_ (μM)		
		A549	HCT116	HepG2
Hymenochirin-1B	48.7	15.22 ± 0.21	12.76 ± 0.43	8.07 ± 0.52
H-1	49.0	10.01 ± 1.21	6.51 ± 0.34	6.32 ± 0.53
H-2	49.3	4.26 ± 0.52	3.54 ± 0.72	1.60 ± 0.24
H-3	65.7	8.67 ± 0.23	4.74 ± 0.71	7.58 ± 0.32
H-4	66.3	24.29 ± 0.41	3.52 ± 0.31	9.98 ± 0.23
H-5	69.2	7.35 ± 0.22	4.16 ± 0.21	2.82 ± 0.23
H-6	64.9	>50	>50	>50
H-7	65.9	8.77 ± 0.22	4.59 ± 0.31	3.04 ± 0.52
H-8	60.5	18.83 ± 0.24	7.72 ± 0.23	25.58 ± 0.32
H-9	64.5	7.87 ± 0.22	3.43 ± 0.35	7.65 ± 0.51
H-10	73.3	3.59 ± 0.12	3.51 ± 0.37	1.50 ± 0.21

### 4. Protease resistance

**Fig. 3:**
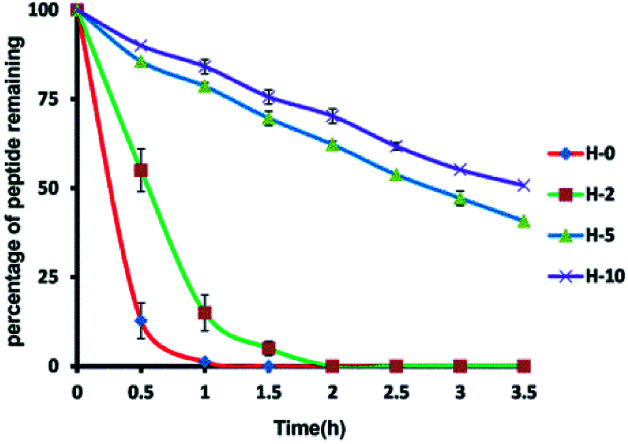
Proteolytic stability of peptides incubated in trypsin solution (5 ng μL^−1^ in 50 mM PBS buffer, pH = 7.4) at a final concentration of 0.1 mM. Date points are displayed as the mean value SEM of duplicate independent experiments. The percent of residual peptide was monitored by Analytic HPLC. All experiments were repeated at least three times. H-0 represents hymenochirin-1B.

To assess the protease stability of the stapled peptides, we measured their susceptibility toward trypsin degradation at room temperature in PBS buffer (pH 7.4) with monitored by HPLC. Trypsin is a protease that predominantly cleaves at the carboxyl terminus of positive charge amino acids, such as arginine and lysine. Under these conditions, the half-life (*t*_1/2_) of hymenochirin-1B is 0.28 h. As expected, hydrocarbon-stapled peptides possessed higher protease resistance, the half-life (*t*_1/2_) of H-2 is 0.5 h, and the half-life (*t*_1/2_) of H-5 is 2.8 h. Especially, only about 49% of bicyclic stapled peptide H-10 was cleaved after 3.5 h (Fig. 3 and S4†). These results demonstrated the obvious superiority of the hydrocarbon-stapled peptide over the linear peptides with respect to protease resistance.

### 5. Growth inhibition of the peptides against tumor cells

**Fig. 4:**
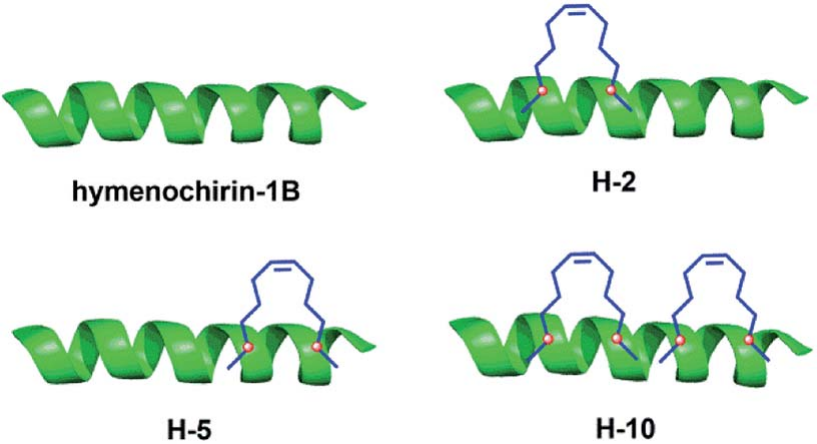
Structures of the linear template peptide hymenochirin-1B, the monocyclic stapled peptides H-2, H-5, the bicyclic stapled peptide H-10. The green represents peptide chain, the blue points to the hydrophobic carbon chains.

The growth-inhibitory activities of peptides were evaluated using the 3-(4,5-dimethylthiazol-2-yl)-2,5-diphenyltetrazolium bromide (MTT) assay against three cancer cells,^35^ namely, human non-small cell lung adenocarcinoma A549 cells, human colorectal adenocarcinoma HCT116 and human adenocarcinoma HepG2. Under the assay condition used in this study, most of stapled peptides maintained or enhanced growth-inhibitory activity to 1.0–5.4 folds than hymenochirin-1B. Among them, the bicyclic stapled peptide H-10 (Fig. 1 and 4) exhibited the best anti-tumor activity compared to the parent peptide on A549 (3.59 μM), HCT116 (3.51 μM) and HepG2 (1.50 μM) with the largest helicity and proteolytic stability than the others. In spite of the helicity of H-2 was only 49.3% and the protease stability was weaker than H-5, H-2 showed higher growth-inhibitory activities against cancer cells than H-5. It might be due to the increase in net positive charge, which the negative residue glutamate was replaced, and the hydrophobic stapled group contributed to effectively interacting with tumor cells in H-2 (Table 1 and Fig. 3).

Meanwhile, H-6 and H-8 showed lower growth-inhibitory activities against cancer cells than the template peptide. According to the study of the peptide sequence, we speculated that it maybe due to one or two lysine residues were replaced in H-8 and H-6, respectively. As a result, the net positive charge of whole peptides was decreased. As previously reported, an increase in the positive charge favored electrostatic interaction of peptide to the negative charged components of membranes, thus enhancing the growth-inhibitory activity against cancer cells. To a certain extent, the net positive charge promoted the growth-inhibitory activity against cancer cells, which may explain that why H-6 and H-8 have lower activities than the parent peptide.

Unlike hymenochirin-1B and H-1, H-5 displayed considerably enhanced activity against three tumor cells (Table 1 and Fig. 4). The results may demonstrate that a higher helicity of peptide is more beneficial for its activity. H-4 and H-6 did not play good roles in tumor cells growth-inhibitory activity, which may be the size and site of macrocyclization disturbed the combination between the peptide and cancer cell, therefore hinders their activities. H-9 also did not work well. A reasonable explanation is that the helix-interrupting glycine residues in hymenochirin-1B allow for the formation of a kinked structure, creating a toroidal pore through the membrane.^4–6,9,30^

To further research the selectivity of these peptides towards cancer cells over normal cells. The growth-inhibitory activities of peptides were evaluated using the 3-(4,5-dimethylthiazol-2-yl)-2,5-diphenyltetrazolium bromide (MTT) assay against three normal cells,^35^ namely, human normal liver cell LO-2, human normal Lung cell BEAS-2 and human normal kidney cell 293 T (Table S2†). The experiments procedure was performed just same as growth inhibition of the peptides against tumor cells. The results showed stapled peptides increase the anti-tumor activity but also increase in normal cells toxicity, which is commonly observed among hydrocarbon stapled peptides.^36–38^ It might be due to the higher averaged hydrophobicity produced by six or nine additional methylene and two olefinic carbons to the peptide to increase toxicity to human normal cells. This situation may be resolved through the addition of polar character and glycosylation into the sequence to decrease the averaged hydrophobicity to improve the selectivity of these peptides towards cancer cells over normal cells. These modifications of these peptides are under way, and the present study was focused on the improved anti-tumor activity of template peptide hymenochirin-1B.

## Conclusions

In summary, ten stapled peptide analogs of hymenochirin-1B were efficiently prepared using a Fmoc-SPPS procedure with Grubbs' first-generation catalyst. Our data demonstrated that the peptide stapling as a conformation constraining strategy can improve the helicity, proteolytic stability and tumor cell-killing activity of α-helical linear peptide hymenochirin-1B in A459, HCT116 and HepG2 cancer cells. Most stapled peptide analogues possessed improved activities against a range of tumor cell lines, in particular, the bicyclic helical peptide H-10 showed a promising prospect for novel anti-tumor drug development. A detailed mechanism study of hymenochirin-1B and its analogs on tumor growth inhibition is currently underway.

## Conflicts of interest

The authors declare no conflict of interest.

## Supplementary Material

RA-008-C8RA03446J-s001
