# Supplementary material for: Stapling strategy enables improvement of antitumor activity and proteolytic stability of host-defense peptide hymenochirin-1B
Source: RSC Adv. 2018 Jun 19;8(39):22268–75. doi: 10.1039/c8ra03446j (PMC9081086; doi:10.1039/c8ra03446j)
Supplement: RA-008-C8RA03446J-s001 [file RA-008-C8RA03446J-s001.pdf]

Electronic Supplementary Material (ESI) for RSC Advances.
This journal is © The Royal Society of Chemistry 2018

Supporting Information

Stapling strategy enables to improve antitumor activity and proteolytic stability of host-defense peptide hymenochirin-1B

Yulei Li[a, b], Minghao Wu[a], Qi Chang[a,b], and Xia Zhao*[a,b]

E-mail: 1184748799@qq.com

Table S1. Electrospray MS data for peptides (positive mode)

Peptide	Molecular Weight (g/mol)	Found Mass	Yield
hymenochirin-1B	3061.87	$[M+2H]^{2+}$ = 1532.4541; $[M+3H]^{3+}$ = 1021.9723	31.23%
H-1	3100.97	$[M+2H]^{2+}$ = 1551.9954; $[M+3H]^{3+}$ = 1034.6734	11.43%
H-2	3069.96	$[M+2H]^{2+}$ = 1535.9828; $[M+3H]^{3+}$ = 1024.3264	6.43%
H-3	3183.03	$[M+2H]^{2+}$ = 1593.0213; $[M+3H]^{3+}$ = 1062.3504	6.51%
H-4	3227.04	$[M+3H]^{3+}$ = 1076.6888; $[M+4H]^{4+}$ = 807.7680;	6.72%
H-5	3170.97	$[M+3H]^{3+}$= 1057.9979; $[M+4H]^{4+}$ = 793.7491	19.94%
H-6	3098.91	$[M+2H]^{2+}$= 1550.9557; $[M+3H]^{3+}$ = 1033.9745	7.25%
H-7	3127.93	$[M+2H]^{2+}$= 1586.9935; $[M+3H]^{3+}$ = 1057.9994	13.07%
H-8	3113.92	$[M+3H]^{3+}$= 1038.9788; $[M+4H]^{4+}$ = 779.4843	14.06%
H-9	3199.99	$[M+3H]^{3+}$= 1067.3436; $[M+4H]^{4+}$ = 800.7592	18.47%
H-10	3177.05	$[M+2H]^{2+}$ = 1590.5354; $[M+3H]^{3+}$ = 1060.3598	5.43%

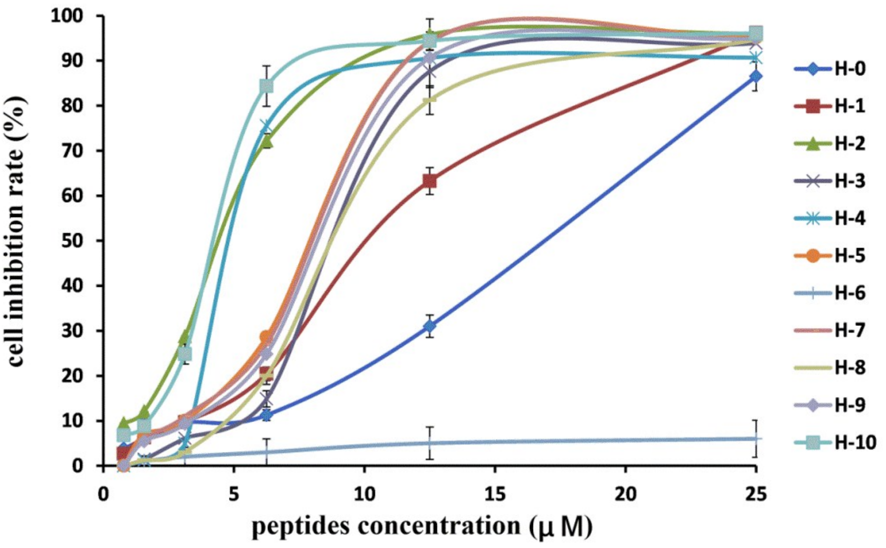

Figure S1. The dose response curves for stapled peptides against non-small cell lung adenocarcinoma A549. H-0 represented template peptide Hymenochirin-1B. All experiments were repeated three times. H-0 represents hymenochirin-1B.

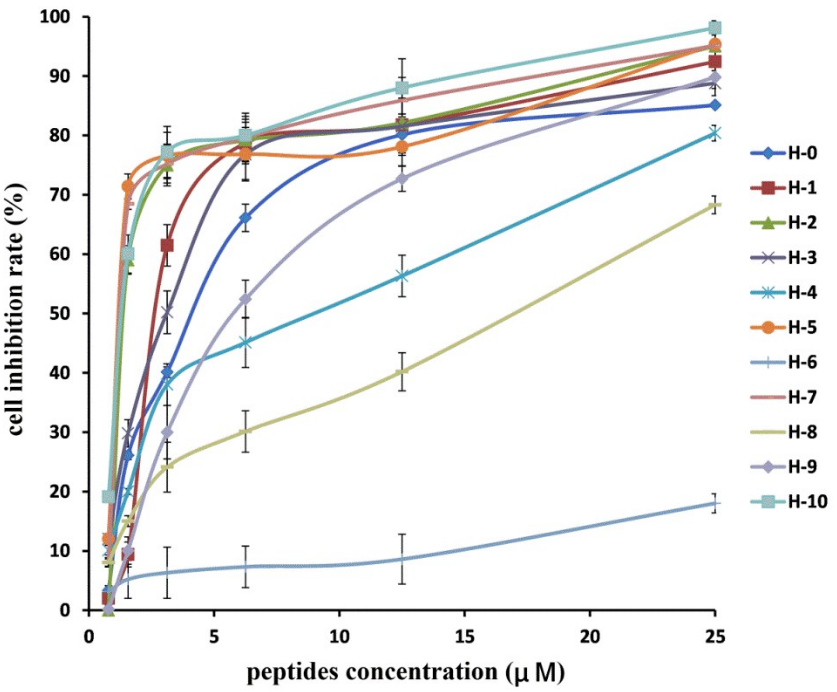

Figure S2. The dose response curves for stapled peptides against hepatocarcinoma HepG2. H-0 represented template peptide Hymenochirin-1B. All experiments were repeated three times. H-0 represents hymenochirin-1B.

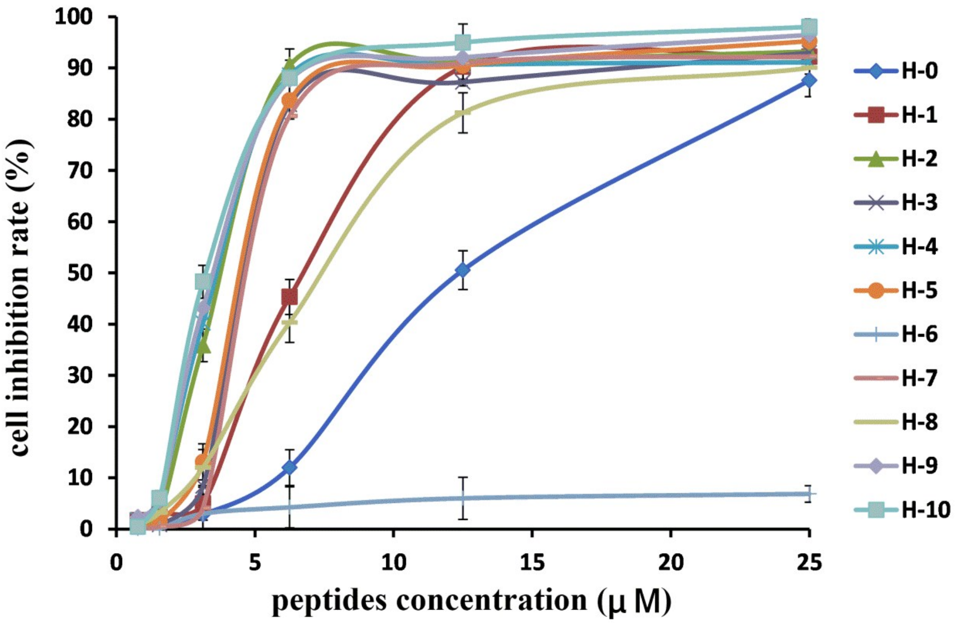

Figure S3. The dose response curves for stapled peptides against colon cancer cell HCT116. H-0 represented template peptide Hymenochirin-1B. All experiments were repeated three times. H-0 represents hymenochirin-1B.

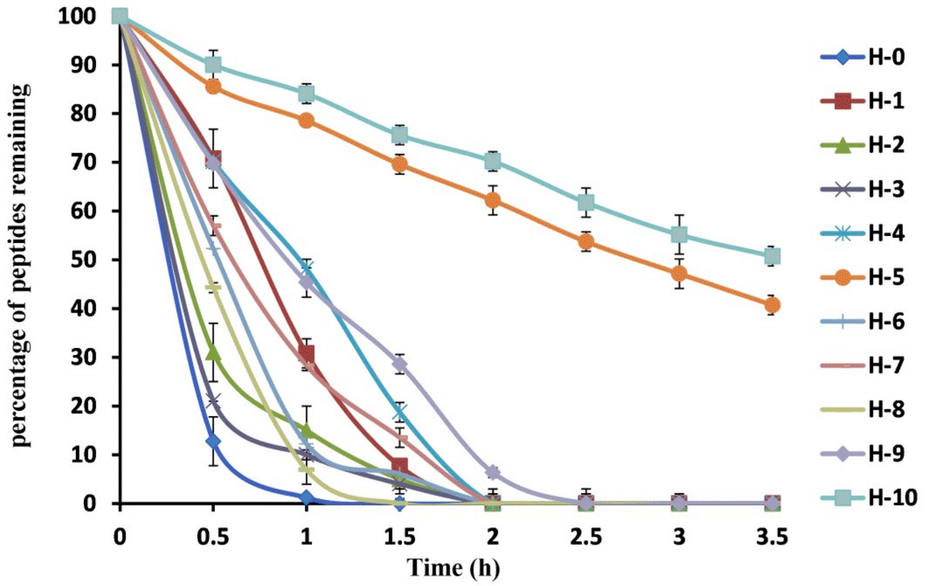

Figure S4. Proteolytic stability of peptides incubated in trypsin solution (5 ng/μl in 50 mM PBS buffer, pH=7.4) at a final concentration of 0.1 mM. Date points are displayed as the mean value SEM of duplicate independent experiments. The percent of residual peptide was monitored by HPLC. All experiments were repeated three times. H-0 represents hymenochirin-1B.

Table S2. Toxicity of the peptides against normal cells

peptide	IC_{50}(μM)		
	**LO-2**	**BEAS-2B**	**293T**
**hymenochirin-1B**	**23.98±0.32**	**9.24±0.25**	**11.12±0.35**
**H-1**	**7.80±0.21**	**9.61±0.35**	**5.82±0.45**
**H-2**	**3.40±0.32**	**3.12±0.52**	**3.20±0.33**
**H-3**	**7.61±0.43**	**7.95±0.36**	**4.58±0.34**
**H-4**	**8.36±0.42**	**25.01±0.23**	**5.41±0.32**
**H-5**	**3.57±0.52**	**5.76±0.32**	**4.70±0.32**
**H-6**	**49.77±0.22**	**46.58±0.42**	**60.11±0.35**
**H-7**	**6.52±0.32**	**7.50±0.32**	**7.25±0.26**
**H-8**	**24.82±0.44**	**12.82±0.25**	**15.58±0.36**
**H-9**	**8.56±0.32**	**7.72±0.36**	**8.66±0.23**
**H-10**	**8.78±0.32**	**5.52±0.42**	**10.42±0.25**

Spectrum

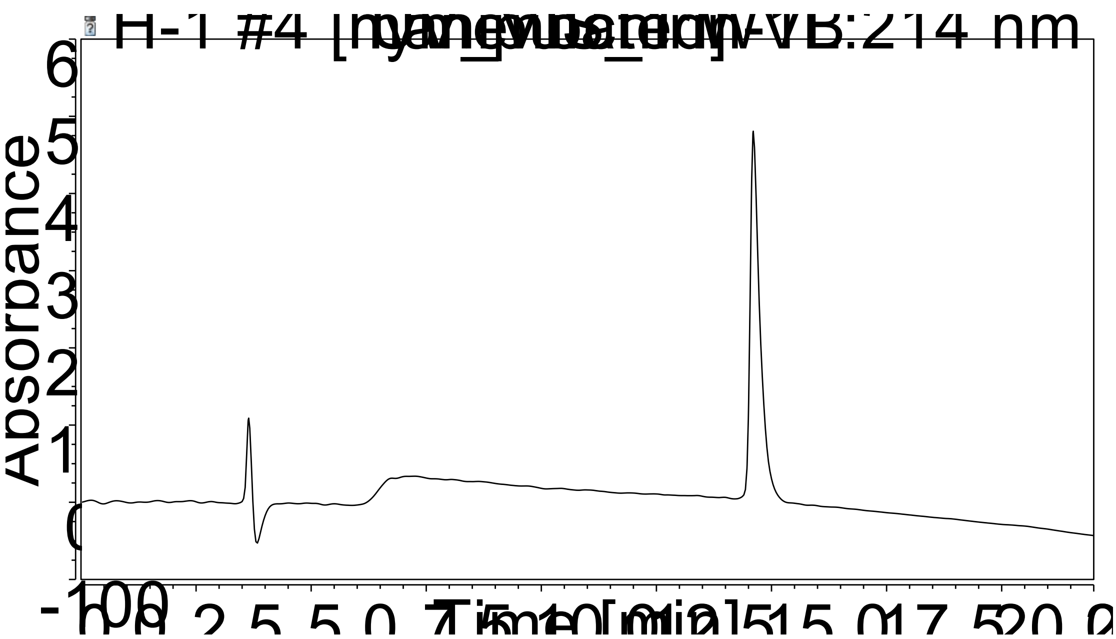

Figure S5. Analytical HPLC trace for compound **hymenochirin-1B**

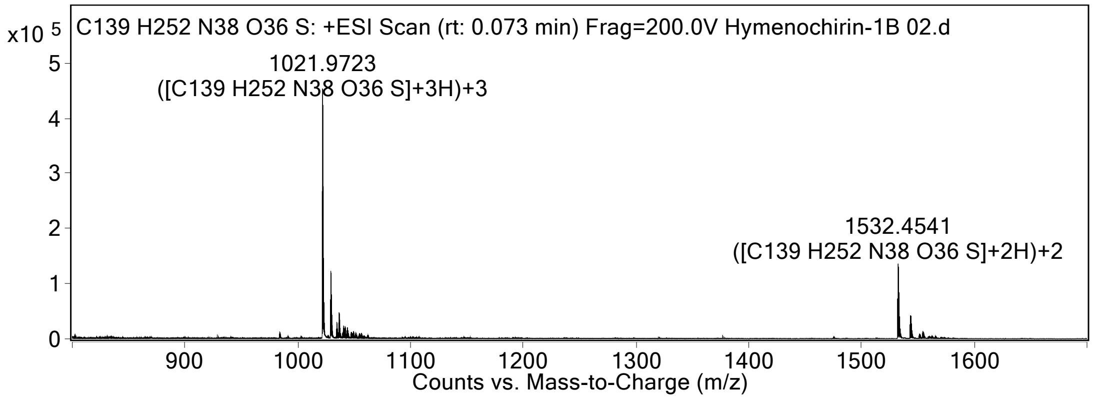

Figure S6. HR-Q-TOF-MS for compound **hymenochirin-1B**

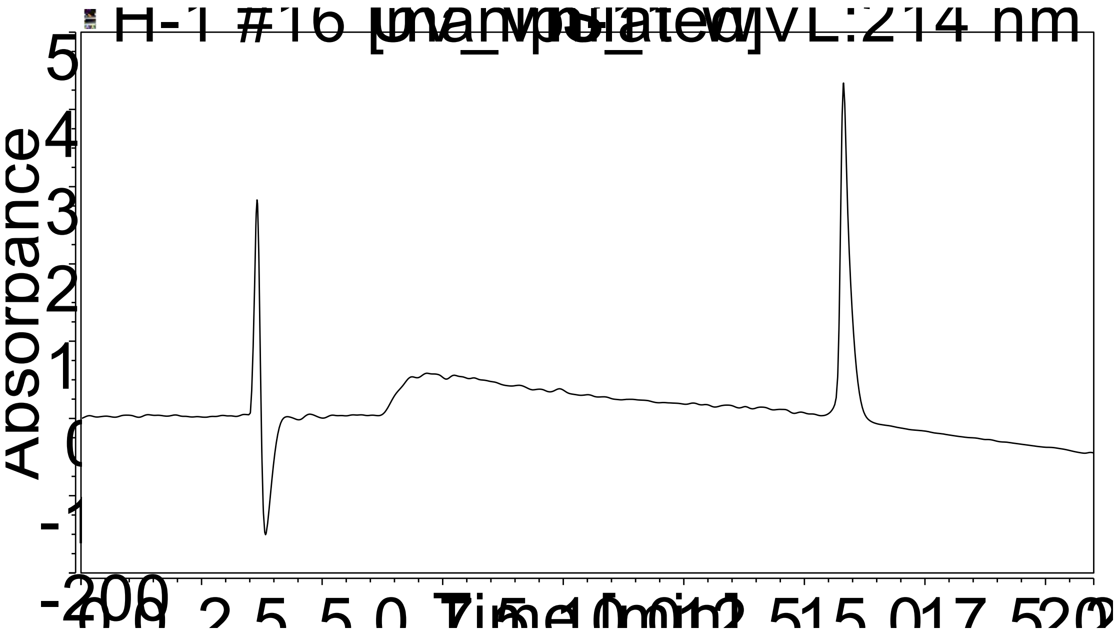

Figure S7. Analytical HPLC trace for compound **H-1**

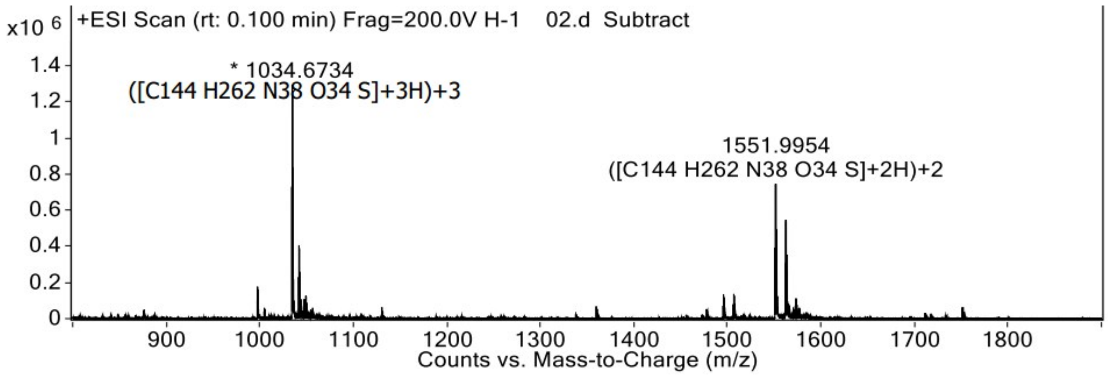

Figure S8. HR-Q-TOF-MS for compound **H-1**

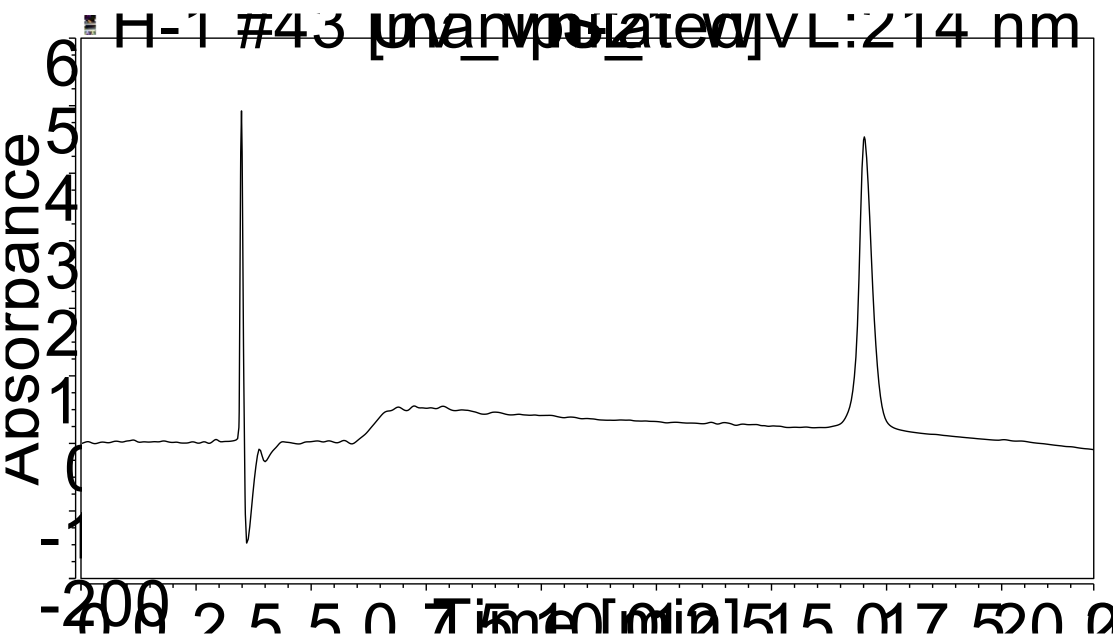

Figure S9. Analytical HPLC trace for compound **H-2**

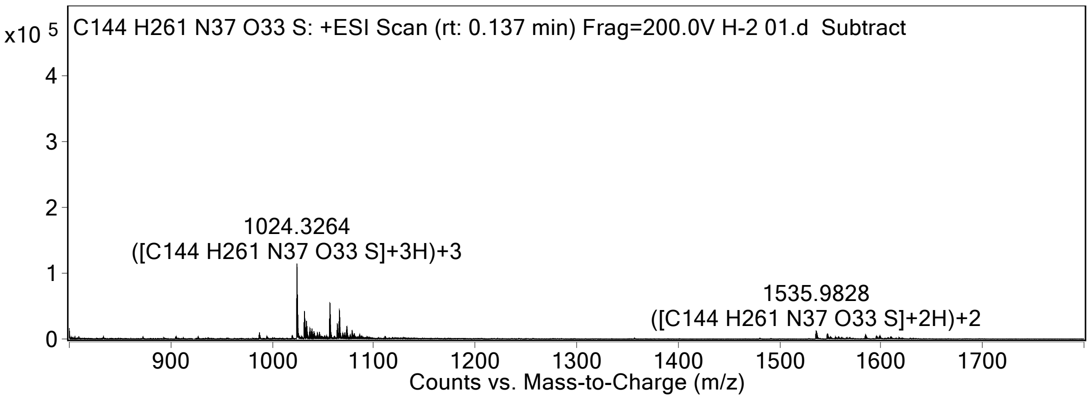

Figure S10. HR-Q-TOF-MS for compound **H-2**

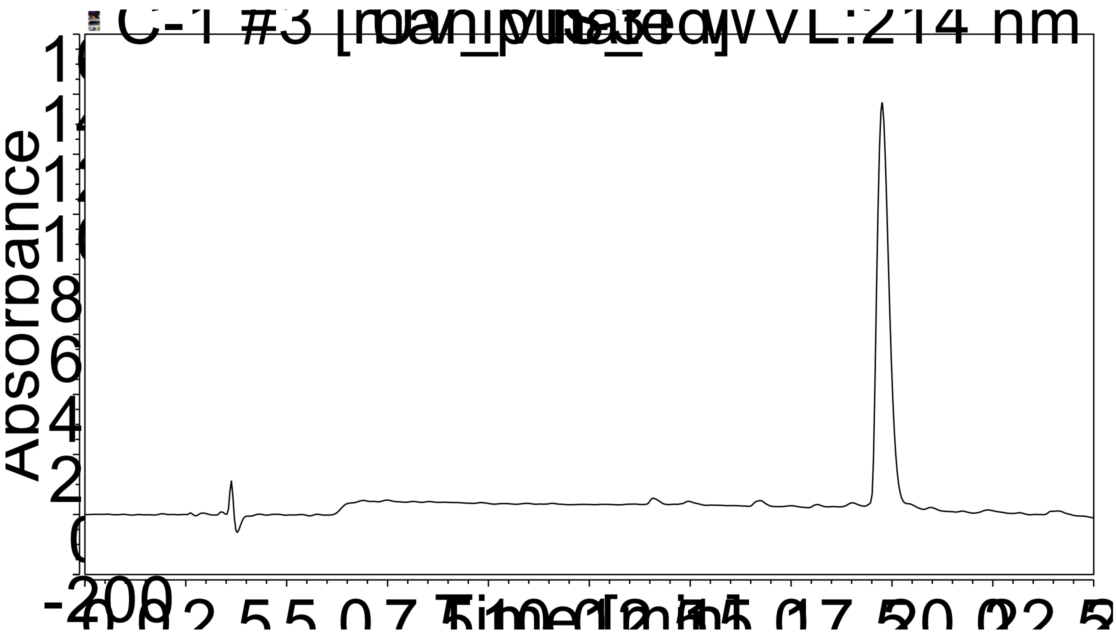

Figure S11. Analytical HPLC trace for compound **H-3**

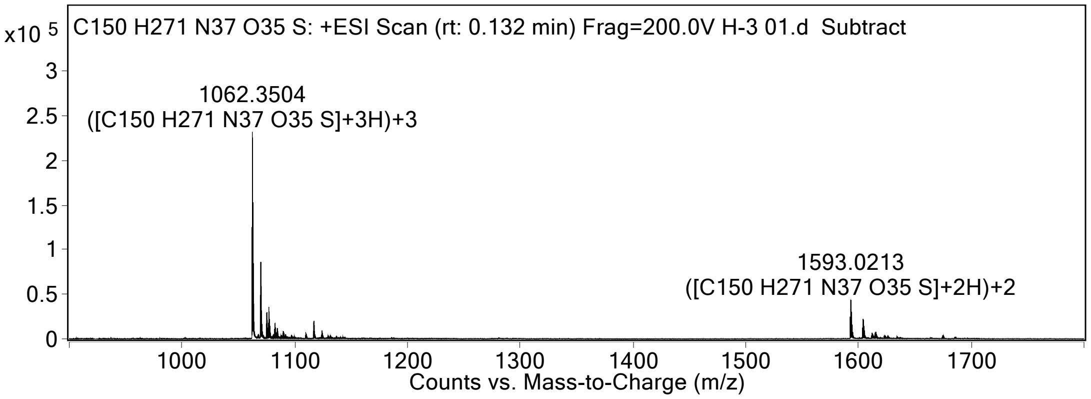

Figure S12. HR-Q-TOF-MS for compound **H-3**

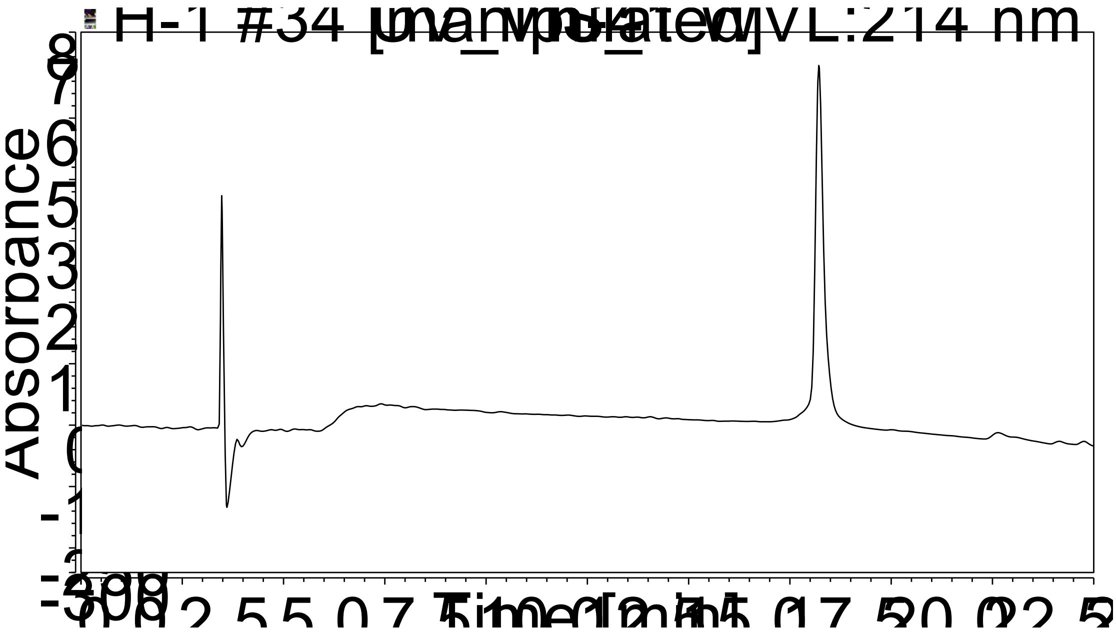

Figure S13. Analytical HPLC trace for compound **H-4**

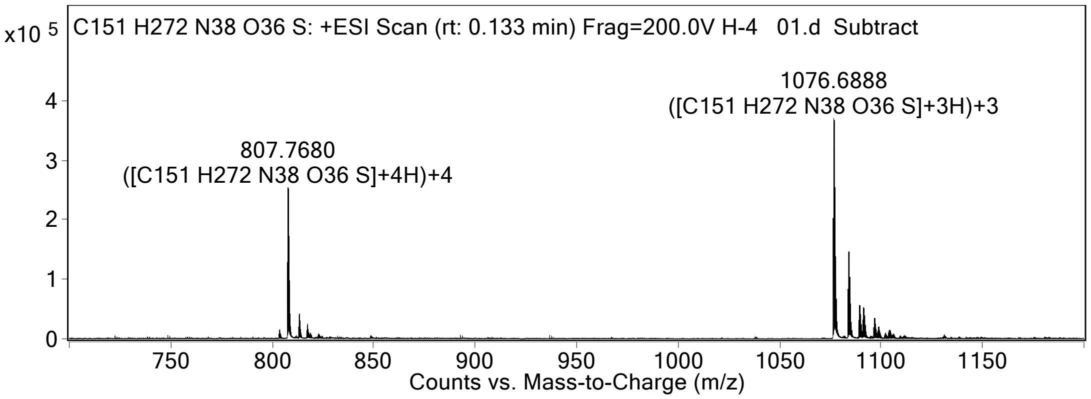

Figure S14. HR-Q-TOF-MS for compound **H-4**

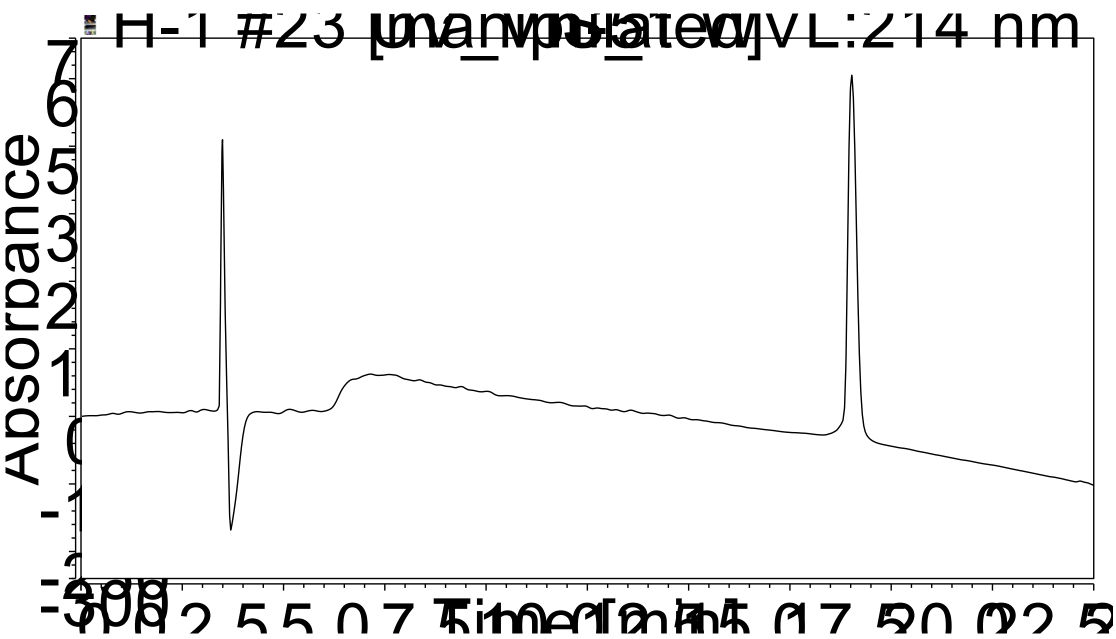

Figure S15. Analytical HPLC trace for compound **H-5**

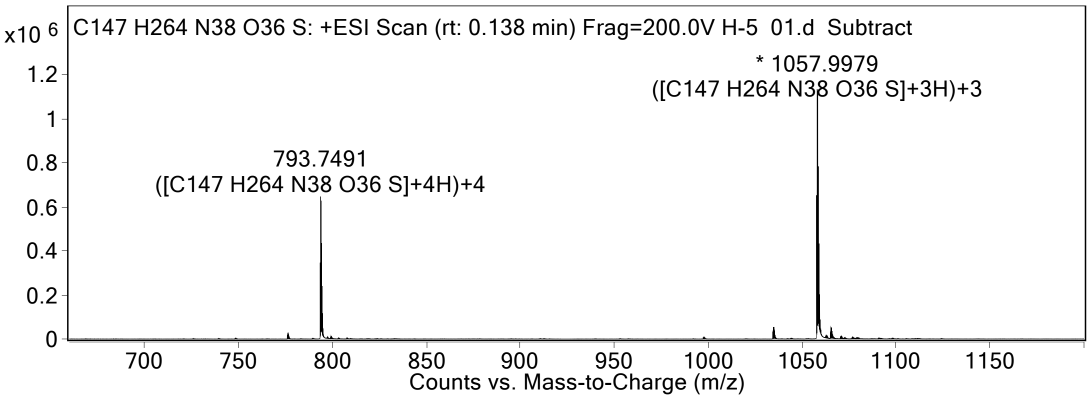

Figure S16. HR-Q-TOF-MS for compound **H-5**

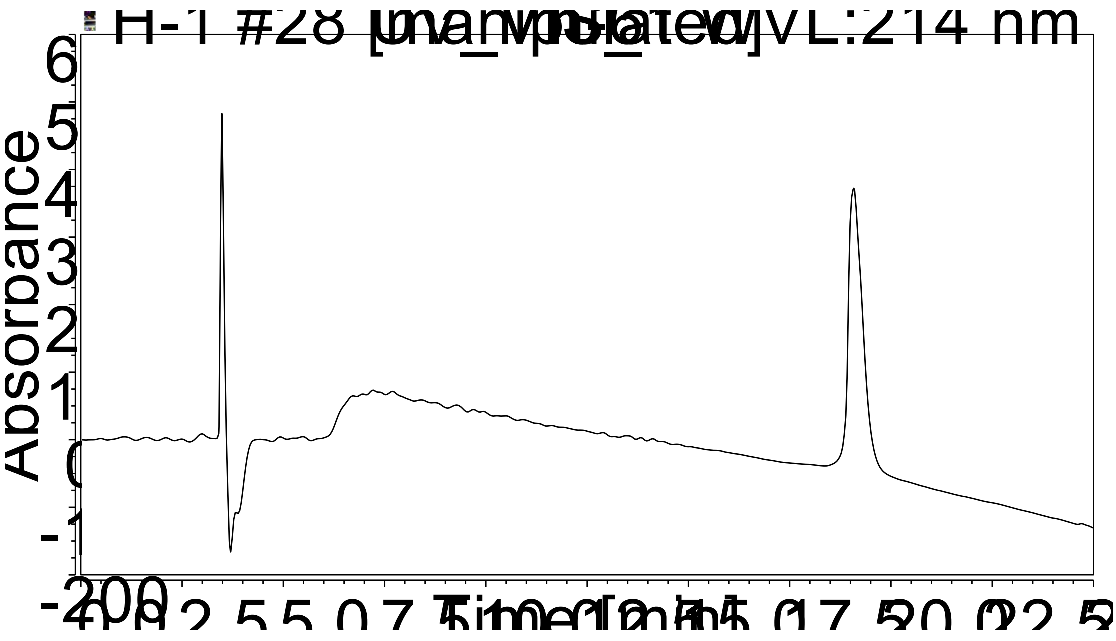

Figure S17. Analytical HPLC trace for compound **H-6**

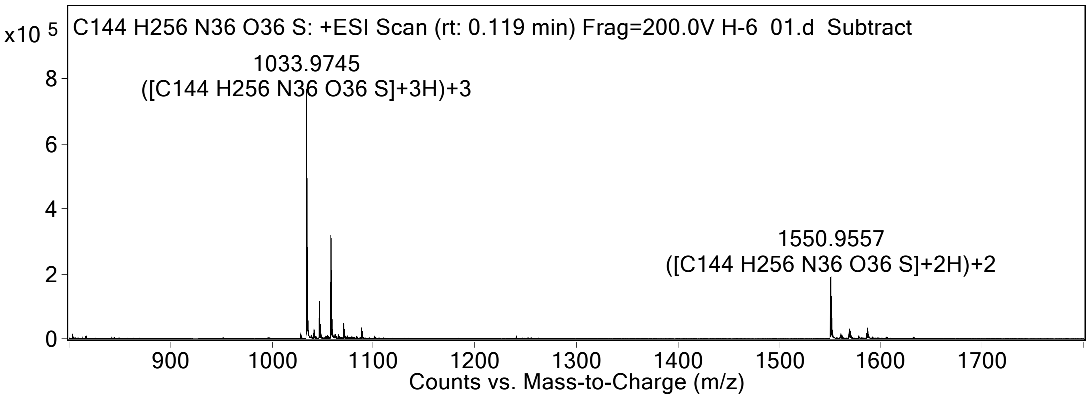

Figure S18. HR-Q-TOF-MS for compound **H-6**

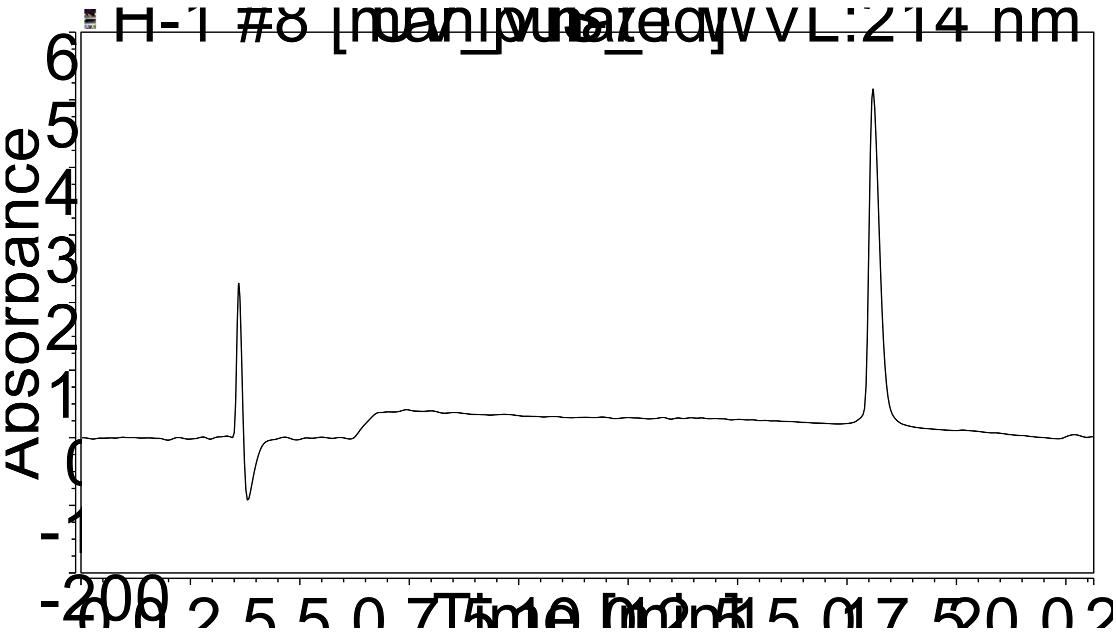

Figure S19. Analytical HPLC trace for compound **H-7**

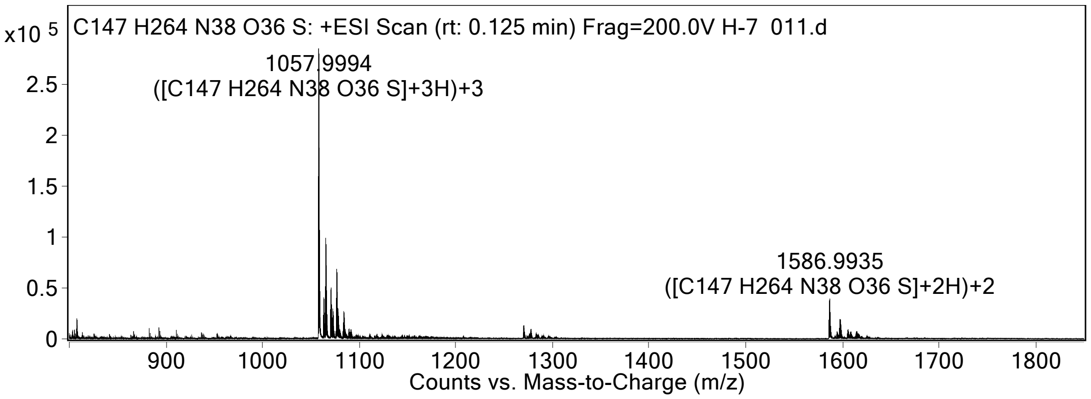

Figure S20. HR-Q-TOF-MS for compound **H-7**

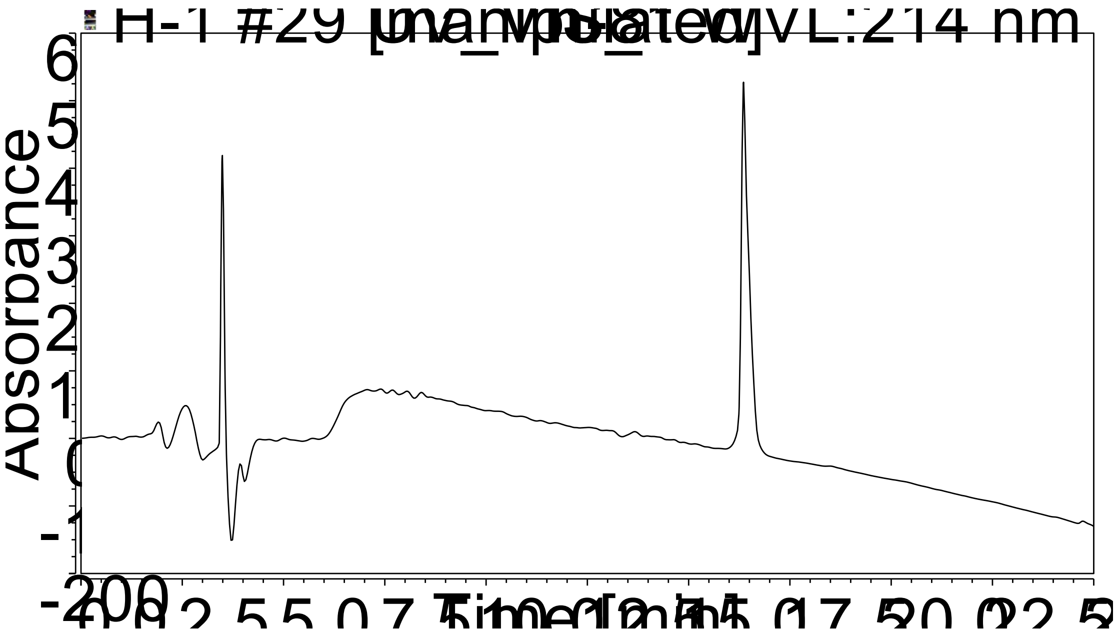

Figure S21. Analytical HPLC trace for compound **H-8**

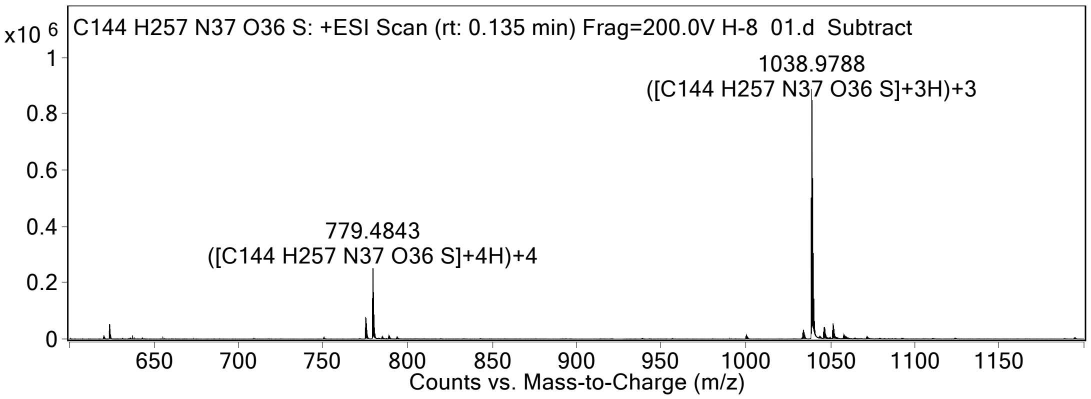

Figure S22. HR-Q-TOF-MS for compound **H-8**

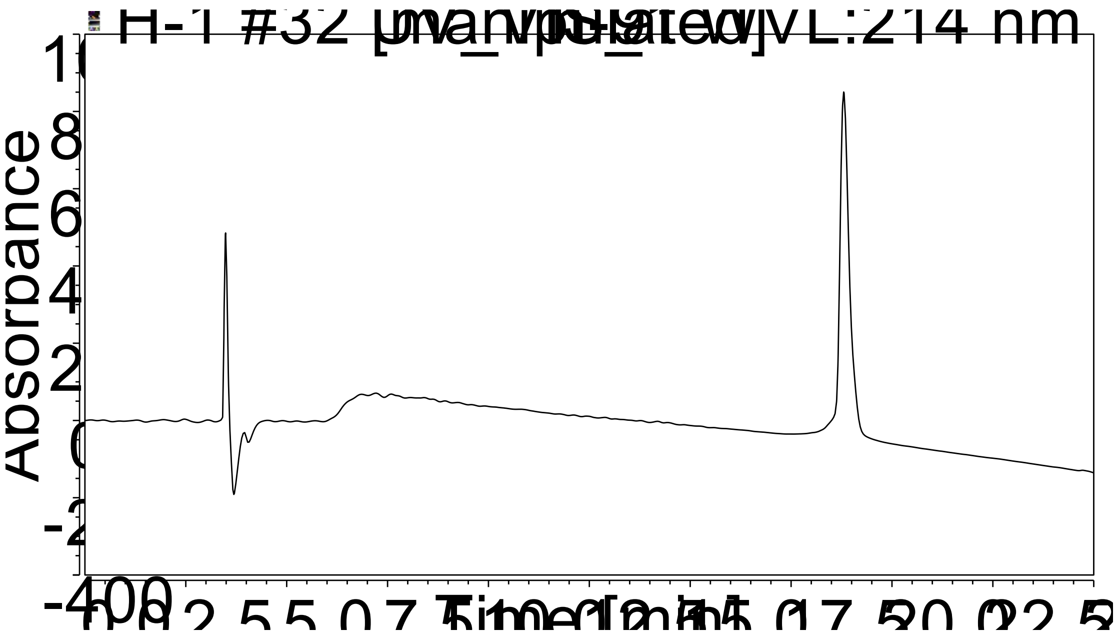

Figure S23. Analytical HPLC trace for compound **H-9**

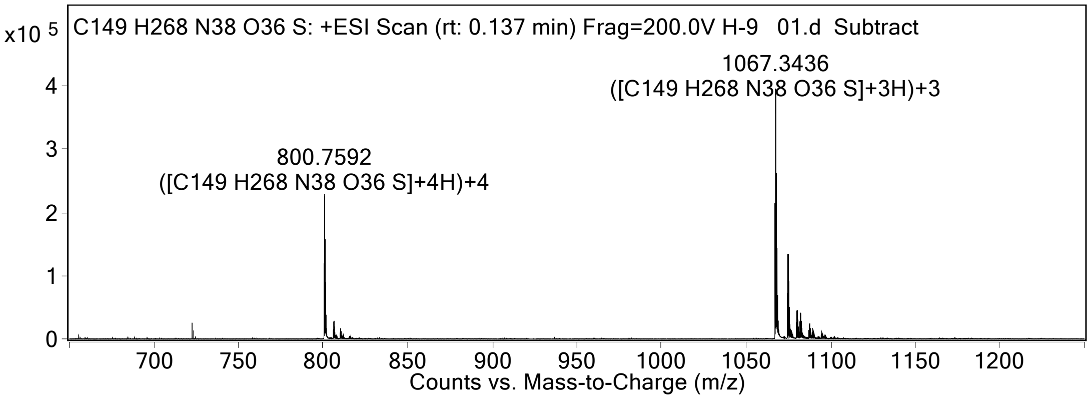

Figure S24. HR-Q-TOF-MS for compound **H-9**

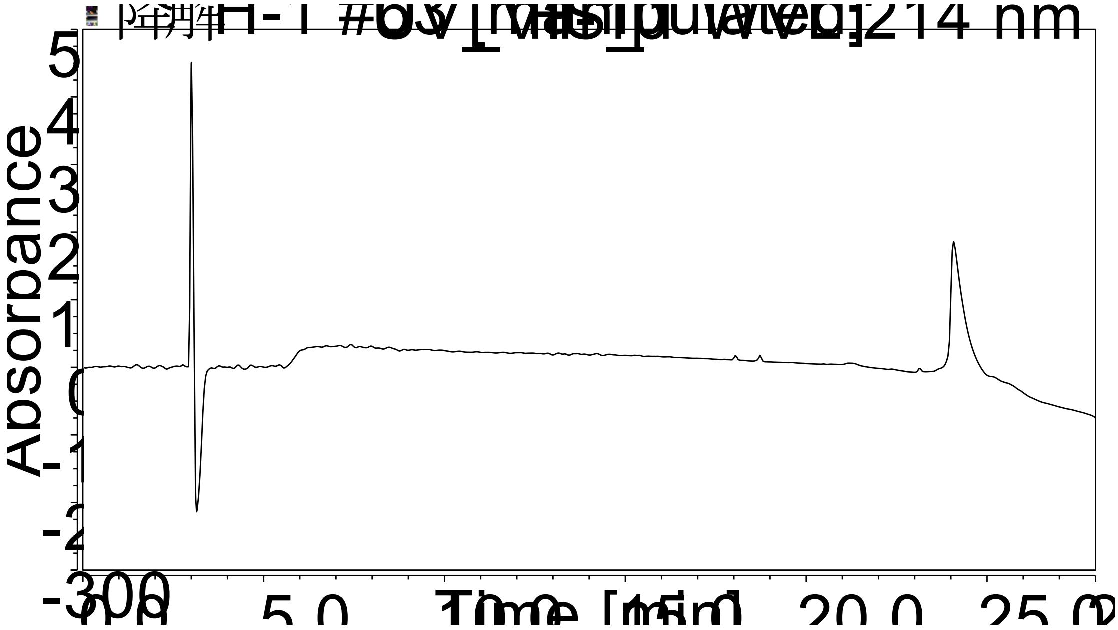

Figure S25. Analytical HPLC trace for compound **H-10**

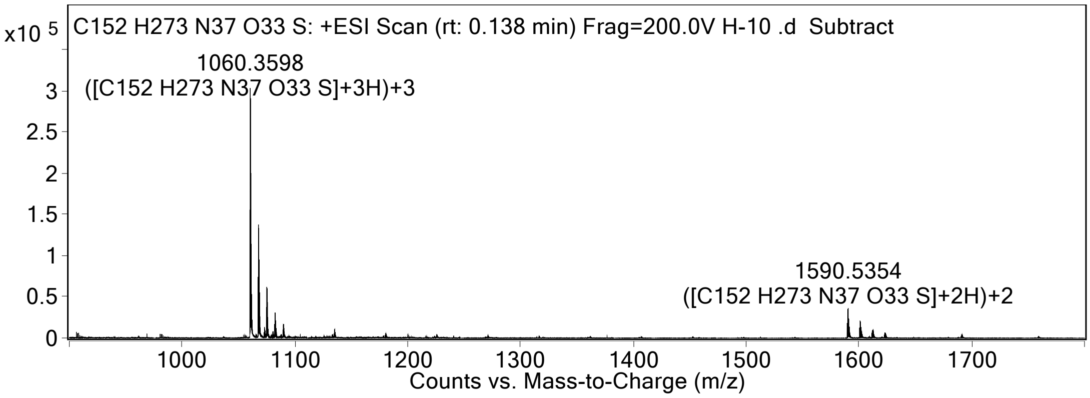

Figure S26. HR-Q-TOF-MS for compound **H-10**
